# Psychometric properties of the Norwegian version of the hospital survey on patient safety culture in a prehospital environment

**DOI:** 10.1186/s12913-018-3576-x

**Published:** 2018-10-17

**Authors:** Leif Inge K Sørskår, Eirik B Abrahamsen, Espen Olsen, Stephen J M Sollid, Håkon B Abrahamsen

**Affiliations:** 1 Institute for Safety, Economics and Planning, University of Stavanger, Kjølv Egelands hus, Kristine Bonnevies vei 22, 4021 Stavanger, Norway; 2 Centre for Resilience in Healthcare, Faculty of Health Sciences, University of Stavanger, Kjell Arholms hus, Kjell Arholms gate 39, 4021 Stavanger, Norway; 3 Faculty of Health Sciences, University of Stavanger, Norway & Prehospital Clinic, Stavanger University Hospital, Stavanger, Norway; 4 Department of Anesthesiology and Intensive Care, Stavanger University Hospital, Stavanger, Norway

**Keywords:** Prehospital, Emergency medical services, Patient safety culture, Patient safety climate, HSOPSC, Psychometric properties

## Abstract

**Background:**

To develop a culture of patient safety in a regime that strongly focuses on saving patients from emergencies may seem counter-intuitive and challenging. Little research exists on patient safety culture in the context of Emergency Medical Services (EMS), and the use of survey tools represents an appropriate approach to improve patient safety. Research indicates that safety climate studies may predict safety behavior and safety-related outcomes. In this study we apply the Norwegian versions of Hospital Survey on Patient Safety Culture (HSOPSC) and assess the psychometric properties when tested on a national sample from the EMS.

**Methods:**

This study adopted a web based survey design. The Norwegian HSOPSC has 13 dimensions, consisting of 46 items, in addition to two single-item outcome variables. SPSS (version 21) was used for descriptive data analysis, estimating internal consistency, and performing exploratory factor analysis. Confirmatory factor analysis (CFA) was applied to test the dimensional structure of the instruments using Amos (version 21).

**Results:**

N = 1387 (27%) EMS employees participated in the survey. Overall, acceptable psychometric properties were observed, i.e. acceptable internal consistencies and construct validity. The patient safety climate dimensions with highest scores (number of positive answers) were “*teamwork within units*” and “*manager expectations & actions promoting patient safety*”. The dimension “*hospital management support for patient safety*” had the lowest score.

**Conclusions:**

The results provided a validated instrument, the Prehospital Survey on Patient Safety Culture (PreHSOPSC), for measuring patient safety climate in an EMS setting. In addition, the explanatory power was strong for several of the outcome dimensions; i.e., several of the safety climate dimensions have a strong predictive effect on outcome variables related to employees’ perceptions on patient safety and safety-related attitude.

**Electronic supplementary material:**

The online version of this article (10.1186/s12913-018-3576-x) contains supplementary material, which is available to authorized users.

## Background

Emergencies appears to constitute the most challenging situations in medicine. Prehospital emergency medical services (EMS) are sometimes called the ‘extended arm of the hospital’ and are characterized by high activity, time pressure, constantly changing environments, and uncertainty; a demanding mix for the providers, and prone to misconduct and errors [1].

Threats to patient safety in the prehospital setting consist of e.g. medication administration errors [2], communication problems [3], deviation from instructions [4], insufficient information [5], lack of training [6], intubation issues [7], patient condition and the related decision-making [8]. Some threats are technical, related to e.g. stretcher issues [9], crash related issues [10] or the introduction of new technology [11]. Frequent handovers between the different EMS organizations may also cause miscommunications and adverse events [12]. Near misses and adverse events appear to be common in the EMS setting, but the culture may suppress the reporting and sharing of such occurrences [1].

Since the famous report *To Err is Human* was published by the Institute of Medicine around the millennium shift [13], the amount of literature on understanding patient safety has grown – but in the context of EMS there is little research on patient safety, and thus little is understood [14, 15]. A literature study [16] pointed to knowledge gaps in the clinical handover of patients arriving by ambulance at the emergency department; e.g. handover information, transfer of responsibility, and staff perceptions and training. Another literature study [17] revealed several gaps compared to the established literature on patient safety, e.g. research into prehospital staffing, safety culture and climate, near-miss reporting, nosocomial infections, quality improvement techniques, and human factors engineering.

For the further improvement of patient safety in health care, safety culture is seen as an important issue and premise [18–20]. A commonly used definition for safety culture is “*the product of individual and group values, attitudes, competencies and patterns of behavior that determine the commitment to, and the style and proficiency of, an organization’s health and safety programmes* (sic)*. Organizations with a positive safety culture are characterized by communications founded on mutual trust, by shared perceptions of the importance of safety, and by confidence in the efficacy of preventive measures*” [21]. Safety culture is developed in response to local conditions, past events, employees’ attitudes, and leadership’s safety-related attitudes and actions. The latter is especially crucial in the development of a good safety culture [1]. It exists several reports on the significant relationship between patient safety culture and specific patient outcomes [22], and improved safety culture is also related to safety performance and a lower incidence of adverse events [19, 23].

Safety climate is a term often used interchangeably with safety culture. Safety climate is commonly defined as “*surface features of the safety culture from attitudes and perceptions of individuals at a given point in time*” [21]. In other words, safety climate research is a ‘snapshot’ of the safety culture, and hence has less depth and is more transient than safety culture. Safety climate research concerns subjective perceptions and attitudes relating to a phenomenon and should not be mistaken for an objective view of the same phenomenon [24]. Safety behavior has been found to have a strong association with safety climate [25–27]. Research indicates that safety climate studies may predict safety behavior and safety-related outcomes such as harm or accidents [28]. Safety climate assessments have become a common practice in health care organizations, and the purposes are e.g. to conduct safety benchmarks and evaluate trends, to identify, monitor and proactively manage safety issues, to evaluate initiatives and interventions, and to meet regulatory requirements [18, 25, 29]. Such assessments have been made for over two decades, and a growing number of studies report on their value [23, 30].

Several instruments have been developed to assess patient safety climate in health care services [21]. Survey methods are regarded as a good way to study attitudes, values and perceptions, and this appears to be the dominant approach for assessing safety climate [31]. One of these is the Hospital Survey on Patient Safety Culture (HSOPSC), which was originally developed by the Agency for Healthcare Research and Quality (AHRQ) for use in hospitals. The dimensions of HSOPSC were chosen based on a literature review of the research, with a focus on safety, errors and misconducts, and on the existing instruments for measuring safety climate [32]. The HSOPSC has several positive attributes; it is one of the few safety climate measuring instruments in which initial psychometric properties are reported, it is designed for both clinical and non-clinical personnel, it distinguishes between organizational- and unit-level, there is increased use in different countries and contexts, and measuring the frequency of reported unwanted events may collaborate well with an organization’s wish for a better reporting climate [33].

Previous studies in Norway have examined the applicability of this instrument in a Norwegian setting, and the Norwegian translation has been validated for the hospital sector [34–36], nurses in intensive care units [37], and in an operating theatre setting [38]. However, applying the instrument in a prehospital setting would interfere with the contextual meaning of the items, affected by e.g. management style, team organization and tasks, and the implementation of reporting systems. The dimensions measured by the instrument, and the underlying model of patient safety climate may be incomplete, only partly applicable for the EMS setting. This requires a new test of the psychometric properties of the instrument in a prehospital context. There is a continued need for research into psychometric properties and the reliability and validity of replicated instruments [33, 34, 38–40]. The aim of our study was to test psychometric properties for HSOPSC performed in a prehospital context.

## Method

Our testing of the HSOPSC in a prehospital context may be described as a three-stage process: (1) define the relevant population and retrieve necessary permissions and respondents’ contact information, (2) pre-test and adjust the instrument, (3) perform data collection and statistical analysis.

### Population characteristics

Regional health trusts are responsible for the Norwegian EMS activities. Their main task is to maintain a state of medical emergency preparedness outside the hospitals and provide transport where acute medical treatment or monitoring is required. In the case of ground EMS (GEMS; car- and boat ambulance), cars are normally staffed by two persons: either two emergency medical technicians (EMT) or one EMT and another licensed health care worker with necessary EMS competence, e.g. a paramedic, a nurse or a physician. For the boat ambulance, the requirement is at least one EMT, in addition to the skipper. Some emergency missions in GEMS may require accompanying healthcare personnel with special medical competence, such as in the transportation of critically ill patients [40]. Norwegian EMTs have a high-school based vocational education, followed by a two-year apprenticeship working as an EMT, before gaining authorization. In addition to the EMT authorization, a paramedic has 60 to 180 European Credit Transfer and Accumulation System (ECTS) points [41]. Supplementing GEMS, helicopter EMS (HEMS) represents the sharp end of the prehospital chain, offering highly competent staff, consisting of an anesthesiologist, a rescuer (HEMS crewmember; HCM), and a pilot. HEMS is vital for providing patients with time-critical medical treatment, particularly in situations involving long distances to the relevant hospital [40]. Search and Rescue Services (SAR) and fixed wing (FW) air ambulances were excluded, since their mission profile and crew concepts differ substantially from HEMS, leading to an exclusion if such personnel were found among the respondents.

### Questionnaire

The Norwegian version of the HSOPSC questionnaire was applied for this study. Prior research has translated the questionnaire into Norwegian and back-translated it by two different professionals [34]. Prior HSOPSC research for Norwegian hospitals [35, 36, 38] found that the outcome variable “*number of events reported*” proved to provide poor correlation with the safety dimensions;to compensate, the outcome dimension “*stop working in dangerous situations*” was amended. This outcome dimension reflects perceived individual safety behavior. It is based on items originally included as part of a questionnaire, called the Norwegian Offshore Risk and Safety Climate Inventory (NORSCI), developed through collaboration between the petroleum industry and various research environments during 2000 [42]. The Norwegian version of the HSOPSC instrument thus has 13 dimensions, 46 items and two single-item ‘outcome’ items [35, 36, 38]. The response format ranges from 1 (disagree strongly) to 5 (agree strongly) on a Likert scale. There are also seven items relating to the respondents’ work characteristics (*work area, geographic location, field of competence, patient contact, work hours, seniority in the prehospital area, seniority in position*).

### Pre-test and adjustments of instrument

As the instrument was applied in a prehospital context, we checked the questionnaire on a test group of seven prehospital healthcare workers to ensure correct terminology. In addition, a prehospital patient safety professional helped in finding discrepancies between the hospital and the prehospital setting. The suggested changes are as listed in Table 1.

**Table 1:** Suggested adjustments of the instrument

Component	Basis for change	Description of change
Interpretation of the term ‘hospital level’	The dimensions in the HSOPSC are divided into three ‘hospital’ level dimensions and seven ‘unit’ level dimensions. The dimensions ‘*handoffs and transitions*’ and ‘*teamwork across units*’ are related to a system of different prehospital units, which, for this context, are better understood as ‘the prehospital chain’.	No change; we find this acceptable, as the intended ‘hospital’ level may be understood as ‘organizational’ level [35], different from the ‘local unit’ level.
Interpretation of the term ‘unit’	To clarify whether the unit should be understood as the local hospital, the local station/base or the working crew.	The term ‘unit’ was substituted with the term ‘local unit’, and ‘local unit’ is explained as ‘localized at same geographic place’.
Interpretation of the term ‘shift changes’ in item H11^a^	The term is related to the in-hospital challenge of transferring responsibility for the patient from one care team to another, which is similar to the transfer of the patient between units in the prehospital chain (e.g. between an ambulance and the hospital).	The term ‘shift changes’ was substituted with ‘patient handover’.
Interpretation of idioms in items A14^a^, C3^a^ and H3^a^	It is embedded in prehospital professions to take ‘shortcuts’ in emergency dispatch situations and work in ‘crisis mode’ at the action site. Also, the expression ‘fall between the cracks’ may be difficult to understand in the context of the prehospital chain.	A minor explanation/example was amended to each of the idioms in the questionnaire.
Interpretation of item A5	The item ‘*staff in this local unit work longer hours than is best for patient care*’, is challenging due regulation by the Working Environment Act [65] and not by the EMS management.	No change; the item is trying to capture a facet of the dimension ‘*staffing*’ and its influence on patient safety, independent of practical underlying causes; i.e. the results may indicate a weakness in the regulations.
Interpretation of items A11 and H2	The items A11 ‘*when one area in this unit gets really busy, others help out*’, and H2 ‘*units in the prehospital chain do not coordinate well with each other*’ were both deemed difficult to interpret in a prehospital context. An emergency dispatcher provides and coordinates the assignments for different vehicles, which is not similar to hospital situations where personnel can move and coordinate more freely between units.	No change; this is arguably of little direct relevance for patient safety but relevant for the latent factor ‘*teamwork within units*’. Emergencies may also exist, where it is possible to offer assistance between vehicles, even if this is not the norm.

*Note*: ^a^The items in full text are found in Table 6

We evaluated whether to include the option of “unknown/not applicable” to all or some of the items, similar to other studies [33, 35, 43]. The outcome variable “*frequency of event reporting*” was especially debated, as the average response may differ from the true (objective) value, and those personnel who do not know the frequency should have the option of stating so. The French HSOPSC study [43] experienced overall low missing score values, except for this outcome dimension (11%). The experience is similar for the German HSOPSC study [33], where items belonging to this outcome dimension have a relatively higher rate of “not applicable” answers than items belonging to the other HSOPSC dimensions. We believe the intention of this outcome dimension, as of other dimensions, is to gain the personnel’s perception of the reality. Therefore, it may be useful to force an answer to the items of this dimension (and other items). Consequently, the option of “unknown/not applicable” was not added, which is in accordance with e.g. the original HSOPSC questionnaire [44].

Considering the aims of the study, we believe it is important to keep the instrument as close to the original Norwegian HSOPSC as possible. Consequently, no items were left out or conceptually changed before distribution of the survey.

### Data collection

E-mail addresses for prehospital personnel in the Norwegian GEMS and HEMS were retrieved from prehospital system leaders. We applied a web-based tool (SurveyXact) to conduct the survey, and an individual link to the questionnaire was distributed by e-mail to all personnel. Data were collected between October and December 2016, and non-responders received up to five reminders before the study was closed.

### Statistical analysis

Psychometric assessment of validation was applied [45, 46] to evaluate the HSOPSC.

#### Construct validity

To determine the degree of fit between the sample and the constructed measurement instrument, a confirmatory factor analysis (CFA) was performed to analyze the construct validity, i.e. an assessment of the relationship between items, and between items and an underlying dimension. Negatively worded items were reversed, and covariation was allowed between the underlying dimensions.

The chi-square test is problematic for assessing model fit for large samples [47] and is thus not reported for this study. For assessing global fit, the following indices were applied: Standardized Root Mean Square Residual (SRMR), Tucker-Lewis Index (TLI), Root Mean Square Error of Approximation (RMSEA) and Comparative Fit Index (CFI). A good fit for RMSEA is a value below 0.5 [48]. Values for TLI and CFI in the 0.90s are generally accepted as guidance values for an acceptable fit, while values above 0.95 reflect a good model fit [48, 49]. It has been suggested to use a two-index strategy by reporting SRMR with one of the fit indices (e.g. CFI or RMSEA), with the guidance criteria CFI > 0.95, SRMR < 0.8 and RMSEA < 0.6 [50]. Guidance values for model fit may prove too strict for complex models with large samples, and the values for TLI and CFI should be reduced accordingly [46]; see Table 2.

**Table 2:** Guidance values for model fit indices

Indices	m ≥ 30
Standardized Root Mean Square Residual (SRMR)	< .08
Tucker-Lewis Index (TLI)	> .90
Root Mean Square of Approximation (RMSEA)	< .07
Comparative Fit Index (CFI)	> .90

*Note*: *m* number of items. Based on [46]

Items providing high loadings on a factor would indicate that they converge to a common point, demonstrating convergent validity for a latent construct. All factor loadings should be statistically significant and at least 0.5 or higher (ideally 0.7 or higher) for standardized estimates [46]. It is not desirable to have several loadings at very high levels, and a range of loadings between 0.6 and 0.9 seems reasonable [45].

Discriminant validity means that individual measured items should represent only one latent construct, and the presence of high cross-loadings potentially indicates a lack of discriminant validity. Inter-correlation between the dimensions was examined by Spearman’s Rho correlation: 0.0–0.25 little or no relationship; 0.25–0.50 fair degree of relationship; 0.50–0.75 moderate to good relationship; >0.75 very good to excellent relationship [51]. MANOVA (multivariate analysis of variance; Wilks’ Lambda) was performed to examine whether the different work characteristics had an overall influence on the overall statistical variance of the HSOPSC dimensions.

To evaluate possible other structures of safety climate dimensions, exploratory factor analysis (EFA) was applied. Varimax rotation was adopted to interpret the factor loadings independently. The latent root criterion (latent root > 1) was applied to identify factors and correlations between measured items [45]. The level for acceptable factor loading was set at ≥ 0.4 [46] and the level for (undesired) cross-loadings at ≥ 0.3. EFA was also forced to extract two factors to examine the grouping of system-level and unit-level dimensions.

To find evidence for criterion-related validity, associations between the safety climate dimensions and the outcome variables are developed by use of linear regression.

#### Internal consistency

Cronbach’s alpha was estimated for the different factors to determine whether they yielded internal consistency and acceptable alpha coefficients between 0.70 and 0.90 [52]. Alpha coefficients may understate reliability [46], but this is relatively inconsequential for practical applications such as meta-analysis [53].

Confirmatory factor analyses (maximum likelihood) were estimated using AMOS 21.0. The other statistical analyses were performed using SPSS 21.0.

#### Ethics approval and consent to participate

Approval was obtained from the Norwegian Social Science Data Services (NSD; project number 45723). The Regional Committee for Medical and Health Research West-Norway (REK west) evaluated this project as “not mandatory to submit” (Ref. number 2015/2249). The participants received information regarding the purpose of the study; they were assured that the digital questionnaires were to be treated in confidence and that no participants could be identified in the published material. Their written consent to participate in the study was given at the start of the survey.

## Results

### Sample characteristics

Individuals participating in the survey totaled 1387 (26% response rate from GEMS and 55% from HEMS; combined, 27% of the total population). The GEMS sample was retrieved from questionnaires conducted in 17 (of 18) health trusts. The sample was considered representative, based on variation in demographic variables, e.g. distribution in professional groups, range in seniority, and geographic location.

For the analyses, only returned questionnaires with all items answered were used. The majority of incomplete questionnaires was discontinued early in the survey, and we evaluated that replacing missing values was not expedient. Excluding responses with missing data (listwise deletion) provided 1154 full responses, consisting of the responses from 1045 GEMS employees and 109 HEMS employees. The sample size coincides with suggested criteria: >200 and at least 10 times the estimated parameters [54].

Of the 1154 respondents, a high number worked directly with patients (98%). As shown in Table 3, the largest professional group was EMTs (47%). Most respondents were from the South-East Regional Health Trust (38%), and the rest were evenly divided among the other three regional health trusts. Respondents were distributed evenly among the other seniority intervals, with a median of at least ten years of seniority.

**Table 3:** Demographic and professional characteristics of the 1154 employees in the study

Characteristics	*N* (%)
Prehospital domain	
GEMS	1045 (90.6)
HEMS	109 (9.4)
Professional group	
EMT	544 (47.1)
Paramedic	260 (22.5)
Nurse EMT	146 (12.7)
Anesthesiologist	56 (4.9)
Nurse	40 (3.7)
HCM	31 (2.7)
Pilot	25 (2.2)
EMT apprentice	24 (2.1)
Other healthcare	22 (1.9)
Administrative	6 (0.5)
Regional health trust	
North	212 (18.4)
Middle	225 (19.5)
West	280 (24.3)
South-East	436 (37.8)
Other	1 (0.1)
Prehospital seniority	
5 years or less	221 (19.2)
6 to 10 years	285 (24.7)
11 to 15 years	230 (19.9)
16 to 20 years	207 (17.9)
21 years or more	211 (18.3)

Notes: *EMT* emergency medical technician. ‘Nurse EMT’ represents nurses with authorization as an EMT. ‘Nurse’ represents nurses without authorization as an EMT. *GEMS* ground emergency medical services, *HEMS* helicopter emergency medical services, *HCM* HEMS crew member

### Descriptive statistics

The mean statistics, standard deviation (SD) and confidence interval (CI) for each of the measurement concepts are presented in Table 4. Among the 1154 respondents, the safety grade was reported as ‘excellent’ by 53 (4.6%), ‘very good’ by 644 (55.8%), ‘acceptable’ by 389 (33.7%), ‘poor’ by 63 (5.5%) and ‘very poor’ by 5 (0.4%). The mean value was observed to be 3.59, where 5 represents ‘excellent’ and 1 represents ‘very poor’. The mean for the ten safety climate dimensions for the HSOPSC ranged from 3.03 to 4.03. The patient safety climate dimensions with highest mean scores, i.e. a higher number of positive answers, were “*teamwork within units*” (4.03) and “*manager expectations & actions promoting patient safety*” (3.79). For the single-item “*number of events reported (last 12 months)*”, 460 (39.9%) had filed no reports, 458 (39.7%) had filed 1-2 reports, 177 (15.3%) had filed 3-5 reports, and 59 (5.1%) had filed 6 reports or more. Overall, variance of items was considered adequate.

**Table 4:** Means, standard deviation (SD), 95% confidence interval (CI) and Cronbach’s alpha coefficients measured by the HSOPSC

Measurement concepts	Number of items	Mean (SD)	95% CI	Alpha		
				This study	Original^a^	Other N studies^b^
Outcome measures – single item						
Patient safety grade	1	3.59 (.69)	3.55 to 3.63			
Number of events reported (last 12 months)	1	1.87 (.89)	1.82 to 1.92			
Outcome dimensions						
Overall perception of safety	4	3.73 (.76)	3.68 to 3.77	.76	.74	.49-.78
Frequency of error reporting	3	2.82 (.79)	2.77 to 2.86	.80	.84	.75-.83
Stop working in dangerous situations	4	4.06 (.57)	4.02 to 4.09	.77		.63
Safety climate dimensions – unit level						
Manager expectations & actions promoting patient safety	4	3.79 (.81)	3.74 to 3.83	.83	.75	.71-.85
Organizational learning - continuous improvement	3	3.36 (.74)	3.31 to 3.40	.69	.76	.51-.64
Teamwork within units	4	4.03 (.65)	3.99 to 4.07	.78	.83	.74-.77
Communication openness	3	3.54 (.75)	3.49 to 3.58	.75	.72	.61-.68
Feedback and communication about error	3	3.19 (.81)	3.14 to 3.24	.79	.78	.69-.76
Nonpunitive response to error	3	3.44 (.92)	3.38 to 3.49	.81	.79	.60-.67
Staffing	4	3.59 (.75)	3.55 to 3.64	.65	.63	.56-.68
Safety climate dimensions – system level						
Hospital management support for patient safety	3	3.03 (.80)	2.98 to 3.07	.79	.83	.76-.80
Teamwork across units	4	3.64 (.56)	3.61 to 3.68	.64	.80	.65-.73
Handoffs and transitions	4	3.40 (.66)	3.36 to 3.44	.78	.80	.62-.68
Median alpha				.76	.78	.64-.74

*Notes* The mean score of each of the items belonging to the dimension is calculated, and the mean of these is then taken to give the mean score for the dimension. ^a^Retrieved from [43] ^b^Norwegian studies: [34, 37, 38, 55]

### Internal consistency

Cronbach’s alpha coefficients varied from 0.64 (*teamwork across units*) to 0.83 (*manager expectations & actions for promoting patient safety)* for HSOPSC (Table 4). Removing items from the dimensions with alpha value <0.7 proved to provide either no or marginal value increase. Compared with the dimensions of the original HSOPSC (retrieved from [43]), the median coefficients in our study are slightly lower than the original results. The greatest difference from the original is 0.64 vs. 0.80 (*teamwork across units)*. Compared to published Norwegian studies [34, 37, 38, 55], a majority of coefficients in the current study are either within or higher than the interval of previous observed results. The dimension “*staffing*” has also been observed with a low coefficient in HSOPSC studies from other countries [43], and our value of 0.65 seems high, relative to these other studies (ranging from 0.44 to 0.65), including the original (0.63). The dimension “*teamwork across units*” had an observed value of 0.64, which is relatively low compared to both the Norwegian studies (0.65-0.73) [34, 37, 38] and those of other countries (0.59-0.80) [43].

### Construct validity

CFA was applied to determine the model fit of the HSOPSC. Overall, compared to the guidance values in Table 5, it demonstrates good model fit values.

**Table 5:** Model fit

Indices	Guidance values	HSOPSC
Standardized Root Mean Square Residual (SRMR)	< .08	.043
Tucker-Lewis Index (TLI)	> .90	.91
Root Mean Square of Approximation (RMSEA)	< .07 / .05^a^	.043
Comparative Fit Index (CFI)	> .90	.92

*Notes*: ^a^Acceptable / good fit. Guidance values are based on [46, 48]

Factor analyses revealed three items with loadings below 0.5; item H2 (0.41), item A5 (0.43), and item A11 (0.47). The range of the other loading values was 0.56 to 0.87 (Table 6). All the items observed with relative weak factor loading (<0.50) had been pointed out as challenging during the pre-test of the instruments.

**Table 6:** HSOPSC dimensions and items

Dimension / Item		Factor loadings
Manager expectations & actions promoting patient safety		
C1	My manager says a good word when he/she sees a job done according to established patient safety procedures.	.80
C2	My manager seriously considers staff suggestions for improving patient safety.	.87
C3	Whenever pressure builds up, my manager wants us to work faster, even if it means taking shortcuts*. (*Do not follow all procedures, for example, not implement the dual control of drugs prior to administration.)	.57
C4	My local manager overlooks patient safety problems that happen over and over.	.73
Organizational learning - continuous improvement		
A6	We are actively doing things to improve patient safety.	.68
A9	Mistakes have led to positive changes here.	.59
A13	After we make changes to improve patient safety, we evaluate their effectiveness.	.70
Teamwork within units		
A1	People support one another in this local unit.	.82
A3	When a lot of work needs to be done quickly, we work together as a team to get the work done.	.73
A4	In this local unit, people treat each other with respect.	.81
A11	When one area in this unit gets really busy, others help out.	.47
Communication openness		
D2	Staff will freely speak up if they see something that may negatively affect patient care.	.65
D4	Staff feel free to question the decisions or actions of those with more authority.	.78
D6	Staff are afraid to ask questions when something does not seem right.	.72
Feedback and communication about error		
D1	We are given feedback about changes put into place based on event reports.	.66
D3	We are informed about errors that happen in this local unit.	.76
D5	In this local unit, we discuss ways to prevent errors from happening again.	.79
Nonpunitive response to error		
A8	Staff feel like their mistakes are held against them.	.80
A12	When an event is reported, it feels like the person is being written up, not the problem.	.77
A16	Staff worry that mistakes they make are kept in their personnel file.	.71
Staffing		
A2	We have enough staff to handle the workload.	.59
A5	Staff in this local unit work longer hours than is best for patient care.	.43
A7	We use more agency/temporary staff than is best for patient care.	.61
A14	We work in "crisis mode"* trying to do too much, too quickly. (*The experience of workload beyond what should be normal.)	.65
Hospital management support for patient safety		
H1	Hospital management provides a work climate that promotes patient safety.	.78
H8	The actions of hospital management show that patient safety is a top priority.	.84
H9	Hospital management seems interested in patient safety only after an adverse event happens.	.63
Teamwork across units		
H2	Units in the prehospital chain do not coordinate well with each other.	.41
H4	There is good cooperation among units that need to work together.	.64
H6	It is often unpleasant to work with staff from other units in the prehospital chain.	.64
H10	Units in the prehospital chain work well together to provide the best care for patients.	.59
Handoffs and transitions		
H3	Things “fall between the cracks”* when transferring patients from one unit to another. (*For example, patient information is not transmitted, unclear responsibility for tasks and procedures in patient handover.)	.64
H5	Important patient care information is often lost during shift changes.	.71
H7	Problems often occur in the exchange of information across units in the prehospital chain.	.73
H11	Patient handovers are problematic for patients in the prehospital chain.	.65
Overall perception of safety		
A10	It is just by chance that more serious mistakes don’t happen in this local unit.	.72
A15	Patient safety is never sacrificed to get more work done.	.56
A17	We have patient safety problems in this local unit.	.73
A18	Our procedures and systems are good at preventing errors from happening.	.70
Frequency of error reporting		
F1	When a mistake is made, but is caught and corrected before affecting the patient, how often is this reported?	.76
F2	When a mistake is made, but has no potential to harm the patient, how often is this reported?	.75
F3	When a mistake is made that could harm the patient, but does not, how often is this reported?	.75
Stop working in dangerous situations		
A19	I ask my colleagues to stop work when I think the job is being done in a risky manner.	.63
A20	I report dangerous situations when I see them.	.69
B1	My colleagues stop me if I'm working in a dangerous manner.	.79
B2	I stop working if I think it can be dangerous for me or others to continue.	.57

Note: Dimensions and items based on the original HSOPSC [44], except for the dimension “*Stop working in dangerous situations*”, which is based on the Norwegian HSOPSC extension [36] *Idioms expressed by a minor explanation/example in the bracket text following the statements C3, A14 and H3

Although several factor loadings fell below 0.6, none of the factors had more than one value below 0.59. None of the factors had all loadings of high values (>0.80). Following the reasoning that the values should be between 0.6 and 0.9, both versions indicated an overall acceptable convergent validity.

As shown in Table 7, the inter-correlations ranged from 0.18 to 0.68 for the dimensions. Excluding the outcome dimensions, the inter-correlations between the safety climate dimensions ranged from 0.30 to 0.68 (fair to good degree of relationship). No values revealed a very good to excellent relationship between dimensions (>0.75).

**Table 7:** Inter-correlations (Spearman’s Rho) of the HSOPSC dimensions

Dimension	1	2	3	4	5	6	7	8	9	10	11	12
1. Overall perception of safety												
2. Frequency of error reporting	.32											
3. Stop working in dangerous situations	.46	.30										
4. Manager expectations & actions promoting patient safety	.59	.31	.43									
5. Organizational learning - continuous improvement	.58	.40	.42	.57								
6. Teamwork within units	.55	.29	.41	.55	.52							
7. Communication openness	.55	.39	.42	.62	.57	.52						
8. Feedback and communication about error	.55	.47	.39	.60	.63	.48	.68					
9. Nonpunitive response to error	.52	.31	.33	.54	.48	.46	.59	.52				
10. Staffing	.59	.26	.29	.52	.44	.51	.46	.45	.52			
11. Hospital management support for patient safety	.51	.32	.30	.50	.51	.39	.45	.50	.41	.41		
12. Teamwork across units	.45	.21	.36	.45	.38	.41	.42	.38	.35	.37	.41	
13. Handoffs and transitions	.43	.18	.29	.38	.30	.32	.33	.29	.33	.34	.40	.59

*Note*: Correlations are significant at the 0.01 level (2-tailed)

In addition, by utilizing MANOVA, a significant Wilk’s Lambda (*p* < 0.001) was measured for all different employee characteristics, except for “*seniority in position*” (*p* = 0.060). Overall, acceptable discriminant validity is found.

EFA performed on the 46 items provided eight factors with latent root value greater than 1. The results in full are presented in Additional file 1: Appendix 1. The factors captured 56.2% of the total variance of all the items. The dimensions “*Organizational learning - continuous improvement*”, “*Communication openness*”, “*Feedback and communication about error*” and three of four items from “*Manager expectations & actions promoting patient safety*” loaded into factor 1. Dimensions “*Teamwork across units*” and “*Handoffs and transitions*” loaded into factor 2, and “*Staffing*” and “*Overall perceptions of safety*” loaded into factor 3. Of 16 cross-loadings (> 0.3), three cross-loadings were greater than 0.4 and also greater than the loading on its primary dimension: items A18 (“*Our procedures and systems are good at preventing errors from happening*”), D6 (“*Staff are afraid to ask questions when something does not seem right*”) and A2 (“*We have enough staff to handle the workload*”). Item A18 loaded into factor 1 as specified above, item D6 loaded into factor 4 alongside the dimension “*Nonpunitive response to error*”, and item A2 loaded into factor 5 alongside the dimension “*Teamwork within units*”. Two items showed overall loading below 0.4; items A11 (“*When one area in this unit gets really busy, others help out*”) and C3 (“*Whenever pressure builds up, my manager wants us to work faster, even if it means taking shortcuts*”).

EFA was also applied to confirm the second-order two-factor structure for the seven unit-level dimensions and three system-level dimensions. While most dimensions loaded into the designated factor in the postulated model, the dimension “*Hospital management support for patient safety*” loaded into the unit-level factor (loading 0.57), with a cross-loading on the system-level factor (loading 0.39). Of the total variance, 63.4% was captured by these factors. Evidently, we did not find full second-order level factors as in previous published results for HSOPSC [43].

A regression analysis was conducted for each of the outcome variables (Table 8). The safety climate dimensions had an overall positive effect on the outcome variables, except for the “*number of events reported (last 12 months)*”, which revealed negative influence from the safety dimensions. In addition, this dimension had low explanatory power, relative to the other outcome dimensions. The dimensions “*nonpunitive response to error*” and “*teamwork across units*” were both significant for only one outcome variable.

**Table 8:** Regression analysis testing the concurrent validity of HSOPSC

Safety climate dimensions	Outcome variables				
	Patient safety grade	Number of events reported (last 12 months)	Overall perceptions of safety	Frequency of error reporting	Stop working in dangerous situations
Manager expectations & actions promoting patient safety	.12***	-.17***	.15***		.07*
Organizational learning - continuous improvement	.22***		.22***	.13***	.12***
Teamwork within units	.13***		.10***		.11***
Communication openness				.12**	.13***
Feedback and communication about error	.07*		.06*	.31***	
Nonpunitive response to error	-.05*				
Staffing	.09***	-.13**	.24***		
Hospital management support for patient safety	.11***		.08***	.07*	
Teamwork across units					.11***
Handoffs and transitions	.10***	-.12*	.11***		.06*
Explanatory power (R squared)	.46	.03	.59	.26	.29
F-test	98.2***	4.6***	166.9***	42.2***	48.5***

Note: *p < 0.05; **p < 0.01; ***p < 0.001; empty fields are non-significant (*p* > 0.05)

## Discussion

This study produced two major findings. Firstly, the study provided overall acceptable psychometric properties, i.e. acceptable internal consistencies and construct validity. However, there were a few exceptions related to weak loadings for some items. Secondly, the explanatory power was strong for several of the outcome dimensions; i.e., it offers stronger predictions regarding which safety climate dimensions have an effect on which outcome variables. Based on these two findings, we provide the EMS environment with a suitable instrument for assessing the patient safety climate in prehospital settings – the Prehospital Survey on Patient Safety Culture (PreHSOPSC).

### Validity of the PreHSOPSC

The observed Cronbach’s alphas were between the recommended limits of 0.70 to 0.90 for all but three dimensions (0.64, 0.65 and 0.69), but only the dimension “*teamwork across units*” had a relatively low alpha value, compared to those of other studies. EFA pointed towards an eight-factor construct, instead of the 13 dimensions that constitute the Norwegian HSOPSC. However, with a few exceptions, the results indicated acceptable convergent and discriminant validity, and the CFA demonstrated overall good model fit compared to the recommended values. The regression analyses showed that the outcome variables had explanatory power values in the range 0.26 to 0.59 (26-59%), except for the outcome dimension “*Number of events reported (last 12 months)*” at 0.03 (3%). The latter result is consistent with those of other HSOPSC studies [34, 56]. Rather than being a risk indicator for patient safety, this outcome variable serves better as a change measure to monitor the degree of reporting over time [57].

### Implications

The HSOPSC instrument was primarily developed by AHRQ for hospitals [32]. Although the HSOPSC is tested for different contexts within the healthcare system, it is not applicable for all contexts in general. Further research should test and validate the instrument for other safety contexts to obtain a generalized instrument for measuring safety climate. An implication followed by the difference between the prehospital and the hospital context is to test the network of relationships between the variables; i.e. the existence of a “nomological network” [45]. Future research should investigate further the existence of such a network, and more evidence for nomological validity should be produced.

Another topic for future research is to take a closer look at the weak items identified by the CFA and the EFA, especially the items pointed out as challenging during the pre-test of the instrument. Still, post hoc modification, by means of e.g. modification indices and standardized residuals [45], should be carried out sparingly and based on theoretical and practical plausibility (e.g. [58]). The use of the HSOPSC instrument in a new context is a challenge in itself, and, instead of performing adjustments and modifications, the development of a new instrument targeted on an EMS context may be a better solution. In particular re-evaluating the position of the prehospital chain in relation to the unit level and hospital level, as indicated by both the lack of evidence of second-order level factors and the relatively low alpha value of the safety dimension “*teamwork across units*”, compared to other studies. A disadvantage of developing a new instrument is the lack of opportunity to compare it with other studies.

The dimensionality revealed by the EFA may also prove useful if developing a new instrument. Although testing within the prehospital domain, our results are similar to those of European hospital adaptations of HSOPSC, where the original postulated dimensions were not fully identified. Several studies support the factor combination of “*Teamwork across units*” and “*Handoffs and transitions*” [33, 35, 43, 59]. Other studies found a similar factor combination of the dimensions “*Staffing*” and “*Overall perception of safety*” [33, 39, 54]. The factor combination of dimensions “*Communication openness*”, “*Feedback and communication about error*” and “*Organizational learning - continuous improvement*” is similar to the findings of the Swedish version [54] and partly similar to the findings of several other studies [33, 35, 39, 43, 59, 60]. Our findings for the dimension “*Manager expectations & actions promoting patient safety*” added to the factor combination above did not support other European versions (to our knowledge); alongside the other factor combinations, it should be investigated further in future studies.

In adjusting the terminology of the original Norwegian HSOPSC for a prehospital context before performing the survey, the purpose was to perform as few adjustments as necessary and not to change the instrument conceptually. Based on this, the option of answering “unknown/not applicable” was not included for any of the items in the questionnaire. Although such an approach decreases the risk of missing score values for the items, it may increase the risk of missing other valuable data. Some aspects of patient safety may be less relevant for the prehospital domain compared to the hospital domain and, in ‘forcing’ respondents to provide an answer, there is a risk of not capturing items that either require an amended explanation in the survey or should be considered candidates for modification or removal. AHRQ is developing a new version of the HSOPSC, in which one of the concerns they are focusing on is to add a "does not apply/don’t know" response option [61]. Their argument is that respondents do not know how to answer if an item does not apply to them. In such cases, a “does not apply” option is reasonable, and adopting this option in future testing of the PreHSOPSC should be considered. However, a “don’t know” option may lead respondents to believe that they should objectively know how to respond, which may increase the risk of missing score values. If adding this option, the items of the questionnaire should be worded in such a way that they lead the respondent to answer according to their social-cognitive observation and evaluation of the environment.

A contextual challenge within acute healthcare is related to the outcome dimension “*stop working in dangerous situations*” [35]; employees are expected to continue working in order to e.g. rescue a patient. In general, this may follow three lines in this context, with increased risk for either the patient or the critical care provider/team – or for both. This may arise if the chosen approach to providing critical care is considered riskier, relative to alternative approaches. An example of this is to perform a rescue operation with a line from a helicopter in challenging terrain, due to e.g. elevations or tree height, while a possible option is to carry the patient out to a safer pick-up point. Another example is reckless driving of a car ambulance during an emergency response. A different view may be provided on this challenge; that safety and emergent care are not discordant concepts and EMS quality patient care can be administered in a safe manner [62]. Consequently, the results of this outcome dimension should be evaluated with the purpose of increasing safety for both patients and personnel.

Despite an adequate number of respondents, the response rate was at the lower end of satisfactory. One cause may be related to being distributed only digitally and not on paper. The majority of the email addresses were work email addresses, which may have caused technical difficulties in opening the questionnaires. In addition, if internal communication is not performed by email, a number of respondents may not have opened their email account during the sample period. Due to the scattered geographic nature of the prehospital environment, paper distribution would have been rather difficult to perform, but it would probably have increased the number of respondents. Another attribute in the prehospital environment is the embedded ‘fast pace working’ culture, and what is perceived as a time-demanding survey may cause the employee to not start or complete the questionnaire. This may explain why the majority of the respondents that did not complete the survey also stopped relatively early in the questionnaire. Another observation that may be related to this culture is the following; before starting the survey, the respondents were asked to provide their consent to participate – and nearly 200 responded negatively to this. Consequently, shorter surveys such as the Norwegian HSOPSC-Short [35] may be preferable. Another aspect of the low number of respondents, in addition to the health region not participating, may also be a cultural link to undesired ‘outside’ observations or that the survey is not prioritized due to ongoing staffing processes.

The aforementioned new version of HSOPSC (version 2.0) under development by AHRQ is based on some of the same considerations made in this article, e.g. issues regarding the use of idioms, alignment to other contexts, and length of survey [61]. Although the instrument is still mainly developed for hospitals, this article demonstrates the benefit of testing the suggested changes and a new safety climate instrument in the ongoing patient safety climate research in the prehospital domain.

### Limitations

There are limitations to the data, which must be borne in mind. Firstly, as previously mentioned, the response rate was low relative to other HSOPSC studies (e.g. [33, 34, 39, 43, 54]). Low response rate may cause non-response bias, i.e. a discrepancy between the employees that responded and the those that did not.

Secondly, the study was limited to the main transport part of the prehospital environment (GEMS and HEMS), thus excluding other parties more or less linked to the prehospital chain (e.g. emergency rooms or emergency medical communications center). Hence, the safety climate for the full prehospital environment is not fully measured.

Thirdly, the instrument has not been tested for predictive validity, i.e. provided evidence of correlation with an external criterion separated in time [45], e.g. reporting of errors, degree of patient compensation, or other patient safety outcomes. Until the instrument has been tested against other external criteria in the prehospital setting, the impact on the EMS safety climate is not fully known.

## Conclusion

Conducting safety climate research provides an opportunity to identify and address areas for improving patient safety. Often, an improved safety climate is accomplished through a number of interventions, targeting one or more dimensions at a time [21]. Using surveys to measure the current status is a suggested first step [63, 64]. To our knowledge, this is the first systematic study of patient safety climate in a Norwegian EMS environment by use of the HSOPSC. The HSOPSC has been previously validated for a Norwegian hospital setting, but, as the prehospital context is different, it generates a need to test the instrument for psychometric properties. Both threats to patient safety and new patient safety improvements/interventions require effective validated instruments to evaluate their impact on the prehospital patient safety climate. Hence, it is a satisfactory result of this study to provide the prehospital environment with a validated instrument, the PreHSOPSC, for measuring the prehospital patient safety climate. This is beneficial in the continuous work of improving patient safety, as the application of the PreHSOPSC may both indicate and predict safety behavior and safety-related outcomes.

## Additional file

Additional file 1: Exploratory factor analysis of the Norwegian Prehospital Survey of Patient Safety Culture (PreHSOPSC). Rotated component matrix (DOCX 15 kb)

## Abbreviations

AHRQ: Agency for Healthcare Research and Quality
CFA: Confirmatory Factor Analysis
CFI: Comparative Fit Index
CI: Confidence Interval
ECTS: European Credit Transfer and Accumulation System
EFA: Exploratory Factor Analysis
EMS: Emergency Medical Services
EMT: Emergency Medical Technician
FW: Fixed Wing
GEMS: Ground Emergency Medical Services
HCM: HEMS Crew Member
HEMS: Helicopter Emergency Medical Services
HSOPSC: Hospital Survey on Patient Safety Culture
MANOVA: Multivariate Analysis of Variance
NORSCI: Norwegian Offshore Risk and Safety Climate Inventory
PreHSOPSC: Prehospital Survey on Patient Safety Culture
RMSEA: Root Mean Square Error of Approximation
SAR: Search and Rescue Services
SD: Standard Deviation
SRMR: Standardized Root Mean Square Residual
TLI: Tucker-Lewis Index
