# Supplementary material for: Psychometric properties of the Norwegian version of the hospital survey on patient safety culture in a prehospital environment
Source: BMC Health Serv Res. 2018 Oct 17;18:784. doi: 10.1186/s12913-018-3576-x (PMC6192077; doi:10.1186/s12913-018-3576-x)
Supplement: Supplementary file 1 — Exploratory factor analysis of the Norwegian Prehospital Survey of Patient Safety Culture (PreHSOPSC). Rotated component matrix (DOCX 15 kb) [file 12913_2018_3576_MOESM1_ESM.docx]

Exploratory factor analysis of the Norwegian Prehospital Survey of Patient Safety Culture (PreHSOPSC).

Rotated component matrix

| Dimension | Item | 8-factor solution | | | | | | | |
| --- | --- | --- | --- | --- | --- | --- | --- | --- | --- |
|  |  | 1 | 2 | 3 | 4 | 5 | 6 | 7 | 8 |
| Manager expectations & actions promoting patient safety | C1 | .699 |  |  |  |  |  |  |  |
|  | C2 | .709 |  |  |  |  |  |  |  |
|  | C3 |  |  | .362 | .357 |  |  |  |  |
|  | C4 | .466 |  | .375 |  |  |  |  |  |
| Organizational learning - continuous improvement | A6 | .434 |  |  |  |  |  |  |  |
|  | A9 | .475 |  |  | .314 |  |  |  |  |
|  | A13 | .556 |  |  |  |  |  |  | .307 |
| Communication openness | D2 | .489 |  |  |  |  |  |  |  |
|  | D4 | .613 |  |  | .336 |  |  |  |  |
|  | D6 | .452 |  |  | .556 |  |  |  |  |
| Feedback and communication about error | D1 | .540 |  |  |  |  |  | .334 |  |
|  | D3 | .612 |  |  |  |  |  | .327 |  |
|  | D5 | .646 |  |  |  |  |  |  |  |
| Teamwork across units | H2 |  | .407 |  |  |  |  |  |  |
|  | H4 |  | .584 |  |  |  |  |  |  |
|  | H6 |  | .660 |  |  |  |  |  |  |
|  | H10 |  | .424 | .352 |  |  |  |  |  |
| Handoffs and transitions | H3 |  | .660 |  |  |  |  |  |  |
|  | H5 |  | .720 |  |  |  |  |  |  |
|  | H7 |  | .726 |  |  |  |  |  |  |
|  | H11 |  | .663 |  |  |  |  |  |  |
| Staffing | A2 |  |  | .445 |  | .477 |  |  |  |
|  | A5 |  |  | .544 |  |  |  |  |  |
|  | A7 |  |  | .420 | .398 |  |  |  |  |
|  | A14 |  |  | .602 | .382 |  |  |  |  |
| Overall perception of safety | A10 |  |  | .528 |  |  |  |  |  |
|  | A15 |  |  | .552 |  |  |  |  |  |
|  | A17 |  |  | .527 |  |  |  |  |  |
|  | A18 | .417 |  | .411 |  |  |  |  |  |
| Nonpunitive response to error | A8 | .311 |  |  | .680 |  |  |  |  |
|  | A12 |  |  |  | .679 |  |  |  |  |
|  | A16 |  |  |  | .701 |  |  |  |  |
| Teamwork within units | A1 |  |  |  |  | .773 |  |  |  |
|  | A3 |  |  |  |  | .745 |  |  |  |
|  | A4 |  |  |  |  | .773 |  |  |  |
|  | A11 | .338 |  |  |  | .320 |  |  |  |
| Stop working in dangerous situations | A19 |  |  |  |  |  | .762 |  |  |
|  | A20 |  |  |  |  |  | .720 |  |  |
|  | B1 |  |  |  |  |  | .626 |  |  |
|  | B2 |  |  |  |  |  | .687 |  |  |
| Frequency of error reporting | F1 |  |  |  |  |  |  | .783 |  |
|  | F2 |  |  |  |  |  |  | .773 |  |
|  | F3 |  |  |  |  |  |  | .760 |  |
| Hospital management support for patient safety | H1 | .335 |  |  |  |  |  |  | .653 |
|  | H8 |  |  |  |  |  |  |  | .734 |
|  | H9 |  |  |  |  |  |  |  | .710 |

Note: Extraction method: Principal Component Analysis. Rotation method: Varimax with Kaiser Normalization (Latent root). Rotation converged in 8 iterations.
